# Supplementary material for: Early Life Stress Is Associated with Alterations in Lymphocyte Subsets Independent of Increased Inflammation in Adolescents
Source: Biomolecules. 2024 Feb 22;14(3):262. doi: 10.3390/biom14030262 (PMC10968282; doi:10.3390/biom14030262)
Supplement: Supplementary file 1 [file biomolecules-14-00262-s001.zip › biomolecules-2865224-supplementary.pdf]

Supplementary Materials

Supplementary Table S1. Markers used to determine 21 cell subsets using flow cytometry

CELL TYPE	MARKERS
B lymphocytes	CD3- CD19+
Naive B cells	CD3- CD19+ IgD+ CD27-
IgD- memory B cells	CD3- CD19+ IgD- CD27+
IgD+ memory B cells	CD3- CD19+ IgD+ CD27+
Senescent Bcells	CD19+, CD57+
T cells	CD3+ CD19-
Cytotoxic T cells	CD3+ CD19- CD8+ CD4-
Naïve cytotoxic T cells	CD3+ CD19- CD8+ CD4- CD45RA+ CCR7+
Effector (E) cytotoxic T cells	CD3+ CD19- CD8+ CD4- CD45RA+ CCR7-
Central Memory cytotoxic T cells	CD3+ CD19- CD8+ CD4- CD45RA- CCR7+
Effector Memory (EM) Cytotoxic T cells	CD3+ CD19- CD8+ CD4- CD45RA- CCR7-
Helper T cells	CD3+ CD19- CD8-
Naïve Helper T cells	CD3+ CD19- CD8- CD4+ CD45RA+ CCR7+
Central Memory Naïve T cells	CD3+ CD19- CD8- CD4+ CD45RA- CCR7+
Effector Helper T-cells	CD3+ CD19- CD8- CD4+ CD45RA+ CCR7-
Effector memory helper T cells	CD3+ CD19- CD8- CD4+ CD45RA- CCR7-
Senescent Tcells	CD3+, CD57+
Senescent Cytotoxic Tcells	CD3+, CD57+, CD4-, CD8+
Senescent Helper Tcells	CD3+, CD57+, CD4+, CD8-
Regulatory T cells	CD3+, CD19-, CD4+, CD8-, CD25+, CD127-
Natural Killer Tcells	CD3+, CD56+

Supplementary Table S2. Cell subset characteristics, non-transformed

Cell types	Non-adopted	Previously-Institutionalized
**B lymphocytes**		
Mean (SD)	12.2 (5.79)	12.4 (5.97)
Median [Min, Max]	11.1 [1.77, 36.6]	11.8 [0.180, 32.4]
Missing	9 (9.4%)	4 (4.2%)
**Central Memory Cytotoxic T cells**		
Mean (SD)	0.954 (0.801)	1.04 (0.709)
Median [Min, Max]	0.750 [0, 4.50]	0.825 [0, 3.19]
Missing	9 (9.4%)	4 (4.2%)
**Central Memory Naïve T cells**		
Mean (SD)	10.6 (4.72)	11.6 (3.52)
Median [Min, Max]	10.7 [0, 23.7]	11.3 [3.49, 22.1]
Missing	9 (9.4%)	4 (4.2%)
**Cytotoxic T Cells**		
Mean (SD)	26.7 (7.37)	29.2 (7.15)
Median [Min, Max]	25.8 [5.15, 47.8]	29.0 [10.4, 43.6]
Missing	9 (9.4%)	4 (4.2%)
**Effector Cytotoxic T cells**		
Mean (SD)	31.1 (16.2)	36.0 (14.3)
Median [Min, Max]	26.2 [5.93, 73.2]	32.6 [7.48, 82.7]
Missing	9 (9.4%)	4 (4.2%)
**Effector Helper T cells**		
Mean (SD)	6.08 (5.27)	5.78 (3.61)
Median [Min, Max]	4.52 [0.630, 27.2]	4.99 [0.950, 27.2]
Missing	9 (9.4%)	4 (4.2%)
**Effector Memory Cytotoxic T cells**		
Mean (SD)	8.09 (5.15)	11.5 (6.63)
Median [Min, Max]	7.63 [0, 29.7]	9.22 [2.19, 32.1]
Missing	9 (9.4%)	4 (4.2%)
**Effector Memory Helper T cells**		
Mean (SD)	17.2 (8.85)	23.5 (9.46)
Median [Min, Max]	15.4 [0.560, 42.4]	21.7 [4.27, 50.8]
Missing	9 (9.4%)	4 (4.2%)
**Helper T Cells**		
Mean (SD)	65.2 (8.05)	61.9 (7.59)
Median [Min, Max]	65.5 [42.2, 82.8]	62.7 [48.1, 80.3]
Missing	9 (9.4%)	4 (4.2%)
**IgD- memory B cells**		

Mean (SD)	7.75 (7.49)	10.4 (5.99)
Median [Min, Max]	5.70 [0, 38.2]	10.2 [0.430, 30.4]
Missing	9 (9.4%)	4 (4.2%)
**IgD+ memory B cells**		
Mean (SD)	7.25 (5.02)	6.29 (4.56)
Median [Min, Max]	5.81 [0, 24.7]	4.95 [1.24, 30.1]
Missing	9 (9.4%)	4 (4.2%)
**Naive cytotoxic T cells**		
Mean (SD)	59.8 (17.7)	51.5 (16.5)
Median [Min, Max]	64.5 [16.1, 90.9]	52.1 [11.7, 90.0]
Missing	9 (9.4%)	4 (4.2%)
**Naive helper T cells**		
Mean (SD)	65.6 (12.6)	58.7 (13.2)
Median [Min, Max]	67.6 [19.5, 92.8]	59.6 [12.1, 88.0]
Missing	9 (9.4%)	4 (4.2%)
**Naive B cells**		
Mean (SD)	80.1 (11.1)	77.0 (9.33)
Median [Min, Max]	82.4 [40.2, 100]	77.0 [54.3, 93.3]
Missing	9 (9.4%)	4 (4.2%)
**Natural Killer Tcells**		
Mean (SD)	2.17 (1.89)	2.80 (2.03)
Median [Min, Max]	1.55 [0, 8.89]	2.49 [0.0580, 8.89]
Missing	9 (9.4%)	4 (4.2%)
**Senescent B cells**		
Mean (SD)	0.209 (0.150)	0.265 (0.159)
Median [Min, Max]	0.170 [0, 0.790]	0.230 [0.00312, 0.790]
Missing	9 (9.4%)	4 (4.2%)
**Senescent Cytotoxic Tcells**		
Mean (SD)	54.7 (14.6)	60.2 (14.3)
Median [Min, Max]	54.3 [19.5, 100]	60.7 [22.9, 88.8]
Missing	9 (9.4%)	4 (4.2%)
**Senescent Helper Tcells**		
Mean (SD)	18.1 (8.89)	20.4 (10.0)
Median [Min, Max]	16.7 [0, 53.7]	18.1 [3.44, 45.6]
Missing	9 (9.4%)	4 (4.2%)
**Senescent Tcells**		
Mean (SD)	4.33 (4.52)	6.64 (4.14)
Median [Min, Max]	2.65 [0.260, 22.3]	5.60 [0.0880, 18.6]
Missing	9 (9.4%)	4 (4.2%)

**T cells**		
Mean (SD)	70.9 (12.0)	68.1 (14.1)
Median [Min, Max]	73.5 [23.7, 89.9]	71.5 [1.99, 85.5]
Missing	9 (9.4%)	4 (4.2%)
**Tregs**		
Mean (SD)	6.14 (3.85)	7.14 (3.71)
Median [Min, Max]	5.30 [0.820, 16.4]	7.04 [1.63, 19.1]
Missing	9 (9.4%)	4 (4.2%)

Supplementary Table S3. Full model results with covariates in regressions examining the association between ELS and Immune Cell Subsets

Cell Type	Parameter	Est	SE	t-stat	p-value
B lymphocytes	(Intercept)	16.958	4.68	3.623	<.001
	PI	0.687	0.968	0.71	0.478
	Age	-0.498	0.205	-2.427	0.016
	Sex	0.744	0.953	0.78	0.437
	Time of Blood draw (Minutes since midnight)	0.005	0.008	0.608	0.544
	IL6.ln	-0.138	0.542	-0.255	0.799
	CRP.ln	0.629	0.555	1.133	0.259
IgD- memory B cells	(Intercept)	1.073	0.578	1.857	0.065
	PI	0.325	0.119	2.721	0.007
	Age	0.045	0.025	1.761	0.08
	Sex	0.007	0.118	0.057	0.955
	Time of Blood draw (Minutes since midnight)	0	0.001	0.304	0.761
	IL6.ln	0.05	0.067	0.742	0.459
	CRP.ln	-0.079	0.069	-1.148	0.253
IgD+ memory B cells	(Intercept)	1.365	0.412	3.316	0.001
	PI	-0.129	0.085	-1.517	0.131
	Age	0.049	0.018	2.712	0.007
	Sex	-0.115	0.084	-1.367	0.174
	Time of Blood draw (Minutes since midnight)	0	0.001	-0.259	0.796
	IL6.ln	-0.14	0.048	-2.931	0.004
	CRP.ln	0.021	0.049	0.44	0.661
Naive B cells	(Intercept)	100.678	7.828	12.862	<.001
	PI	-1.131	1.618	-0.699	0.486
	Age	-1.191	0.343	-3.473	0.001
	Sex	1.016	1.595	0.637	0.525
	Time of Blood draw (Minutes since midnight)	-0.008	0.013	-0.597	0.551
	IL6.ln	0.215	0.907	0.237	0.813
	CRP.ln	0.888	0.929	0.956	0.34
Senescent B cells	(Intercept)	0.338	0.125	2.707	0.008
	PI	0.08	0.026	3.088	0.002
	Age	-0.01	0.005	-1.914	0.057

	Sex	0.076	0.025	2.977	0.003
	Time of Blood draw (Minutes since midnight)	0	0	2.066	0.04
	IL6.ln	0.016	0.014	1.09	0.277
	CRP.ln	-0.012	0.015	-0.799	0.426
T cells	(Intercept)	56.64	10.502	5.393	0.236
	PI	-3.769	2.171	-1.736	0.079
	Age	1.032	2.14	0.482	0.647
	Sex	0.142	0.46	0.31	0.785
	Time of Blood draw (Minutes since midnight)	0.023	0.017	1.335	0.188
	IL6.ln	2.597	1.217	2.135	0.029
	CRP.ln	-1.882	1.246	-1.51	0.127
Helper T cells	(Intercept)	65.187	6.216	10.488	<.001
	PI	-3.721	1.285	-2.896	0.004
	Age	0.247	0.272	0.907	0.366
	Sex	-2.594	1.266	-2.048	0.042
	Time of Blood draw (Minutes since midnight)	-0.005	0.01	-0.468	0.64
	IL6.ln	-1.027	0.72	-1.427	0.156
	CRP.ln	0.138	0.737	0.187	0.852
Central memory naive T cells	(Intercept)	2.064	3.188	0.647	0.518
	PI	0.332	0.659	0.503	0.615
	Age	0.451	0.14	3.231	0.001
	Sex	1.013	0.649	1.56	0.121
	Time of Blood draw (Minutes since midnight)	0.003	0.005	0.512	0.609
	IL6.ln	0.638	0.369	1.726	0.086
	CRP.ln	-0.316	0.378	-0.836	0.404
Effector memory helper T cells	(Intercept)	-10.757	6.264	-1.717	0.088
	PI	4.408	1.295	3.404	0.001
	Age	1.567	0.274	5.712	<.001
	Sex	0.478	1.276	0.375	0.708
	Time of Blood draw (Minutes since midnight)	0.008	0.01	0.823	0.411
	IL6.ln	1.61	0.726	2.219	0.028
	CRP.ln	-0.143	0.743	-0.192	0.848
Effector helper T-cells	(Intercept)	0.9	0.395	2.28	0.024
	PI	0.015	0.082	0.189	0.85
	Age	0.014	0.017	0.818	0.415
	Sex	0.009	0.08	0.114	0.91
	Time of Blood draw (Minutes since midnight)	0.001	0.001	2.072	0.04
	IL6.ln	-0.077	0.046	-1.68	0.095
	CRP.ln	0.077	0.047	1.641	0.103
Naive helper T cells	(Intercept)	101.77	9.227	11.03	<.001
	PI	-4.502	1.908	-2.36	0.019
	Age	-1.894	0.404	-4.685	0
	Sex	-2.7	1.88	-1.437	0.153
	Time of Blood draw (Minutes since midnight)	-0.013	0.015	-0.898	0.371
	IL6.ln	-0.486	1.069	-0.454	0.65
	CRP.ln	-1.316	1.095	-1.202	0.231

Cytotoxic T cells	(Intercept)	23.799	5.764	4.129	<.001
	PI	2.606	1.192	2.187	0.03
	Age	-0.051	0.253	-0.201	0.841
	Sex	1.803	1.174	1.536	0.127
	Time of Blood draw (Minutes since midnight)	0.005	0.009	0.561	0.576
	IL6.ln	1.232	0.668	1.845	0.067
	CRP.ln	-0.504	0.684	-0.738	0.462
Central memory cytotoxic T cells	(Intercept)	0.171	0.261	0.656	0.513
	PI	0.003	0.054	0.061	0.951
	Age	0.02	0.011	1.787	0.076
	Sex	0.058	0.053	1.094	0.276
	Time of Blood draw (Minutes since midnight)	0	0	0.492	0.623
	IL6.ln	0.015	0.03	0.5	0.618
	CRP.ln	0.027	0.031	0.878	0.381
Effector cytotoxic T cells	(Intercept)	4.396	11.574	0.38	0.705
	PI	3.946	2.393	1.649	0.101
	Age	0.455	0.507	0.898	0.37
	Sex	2.616	2.358	1.109	0.269
	Time of Blood draw (Minutes since midnight)	0.038	0.019	2.041	0.043
	IL6.ln	-1.089	1.341	-0.812	0.418
	CRP.ln	2.113	1.373	1.539	0.126
Effector memory cytotoxic T cells	(Intercept)	2.389	4.629	0.516	0.607
	PI	3.031	0.957	3.167	0.002
	Age	0.402	0.203	1.983	0.049
	Sex	-0.299	0.943	-0.317	0.751
	Time of Blood draw (Minutes since midnight)	-0.001	0.007	-0.07	0.944
	IL6.ln	-0.25	0.536	-0.466	0.642
	CRP.ln	-0.041	0.549	-0.074	0.941
Naive cytotoxic T cells	(Intercept)	93.254	12.948	7.202	<.001
	PI	-6.881	2.677	-2.57	0.011
	Age	-0.899	0.567	-1.586	0.115
	Sex	-2.348	2.638	-0.89	0.375
	Time of Blood draw (Minutes since midnight)	-0.038	0.021	-1.839	0.068
	IL6.ln	1.389	1.5	0.926	0.356
	CRP.ln	-2.212	1.536	-1.44	0.152
Regulatory T cells	(Intercept)	6.852	2.981	2.299	0.023
	PI	0.937	0.616	1.521	0.13
	Age	0.141	0.131	1.079	0.282
	Sex	0.499	0.607	0.823	0.412
	Time of Blood draw (Minutes since midnight)	-0.006	0.005	-1.206	0.229
	IL6.ln	-0.04	0.345	-0.115	0.909
	CRP.ln	-0.309	0.354	-0.874	0.384
Senescent T cells	(Intercept)	0.109	0.449	0.243	0.808
	PI	0.458	0.093	4.941	<.001
	Age	0.006	0.02	0.324	0.746
	Sex	0.226	0.091	2.467	0.015

	Time of Blood draw (Minutes since midnight)	0.002	0.001	3.027	0.003
	IL6.ln	0.066	0.052	1.262	0.209
	CRP.ln	-0.048	0.053	-0.909	0.365
Senescent cytotoxic T cells	(Intercept)	37.232	11.067	3.364	0.001
	PI	3.605	2.288	1.576	0.117
	Age	0.839	0.485	1.73	0.086
	Sex	4.614	2.254	2.047	0.042
	Time of Blood draw (Minutes since midnight)	0.006	0.018	0.363	0.717
	IL6.ln	2.43	1.282	1.896	0.06
	CRP.ln	-0.191	1.313	-0.145	0.885
Senescent helper T cells	(Intercept)	22.687	7.346	3.088	0.002
	PI	3.199	1.519	2.106	0.037
	Age	-0.124	0.322	-0.385	0.701
	Sex	-4.146	1.497	-2.77	0.006
	Time of Blood draw (Minutes since midnight)	-0.003	0.012	-0.233	0.816
	IL6.ln	-0.426	0.851	-0.501	0.617
	CRP.ln	-0.115	0.871	-0.132	0.895
Natural killer T cells	(Intercept)	-0.179	0.424	-0.421	0.674
	PI	0.131	0.088	1.499	0.136
	Age	0.028	0.019	1.521	0.13
	Sex	0.12	0.086	1.388	0.167
	Time of Blood draw (Minutes since midnight)	0.001	0.001	2.048	0.042
	IL6.ln	0.155	0.049	3.148	0.002
	CRP.ln	-0.046	0.05	-0.91	0.364

Supplementary Table S4. Association between ELS and Immune Cell Subsets in CMV Negative Youth

Cell Subset	Est	SE	t-stat	p-value	Cohen's f2
**B lymphocytes**	0.611	2.078	0.294	0.77	0.002
**IgD- memory B cells**	0.103	0.242	0.426	0.672	0.005
**IgD+ memory B cells**	-0.363	0.17	-2.137	0.037	0.063
**Naive B cells**	4.08	3.155	1.293	0.201	0.019
**Senescent B cells**	0.069	0.044	1.566	0.123	0.028
**T cells**	-0.35	4.339	-0.081	0.893	0.001
**Helper T cells**	-7.051	2.603	-2.709	0.009*	0.147
**Central memory naive T cells**	0.465	1.375	0.338	0.736	0.002
**Effector memory helper T cells**	2.793	2.286	1.222	0.227	0.059
**Effector helper T-cells**	-0.157	0.151	-1.042	0.302	0.001
**Naive helper T cells**	-1.355	2.967	-0.457	0.649	0.03
**Cytotoxic T cells**	5.181	2.248	2.304	0.025*	0.099
**Central memory cytotoxic T cells**	0.007	0.124	0.053	0.958	0.002
**Effector cytotoxic T cells**	0.095	3.899	0.024	0.981	0.008
**Effector memory cytotoxic T cells**	0.969	1.572	0.617	0.54	0.008
**Naive cytotoxic T cells**	-0.867	4.505	-0.192	0.848	0.012

**Regulatory T cells**	1.3	1.319	0.986	0.328	0.014
**Senescent T cells**	0.194	0.123	1.578	0.12	0.073
**Senescent cytotoxic T cells**	-4.548	4.198	-1.083	0.283	0.004
**Senescent helper T cells**	0.373	2.353	0.159	0.875	0.001
**Natural killer T cells**	0.325	0.157	2.071	0.043*	0.12

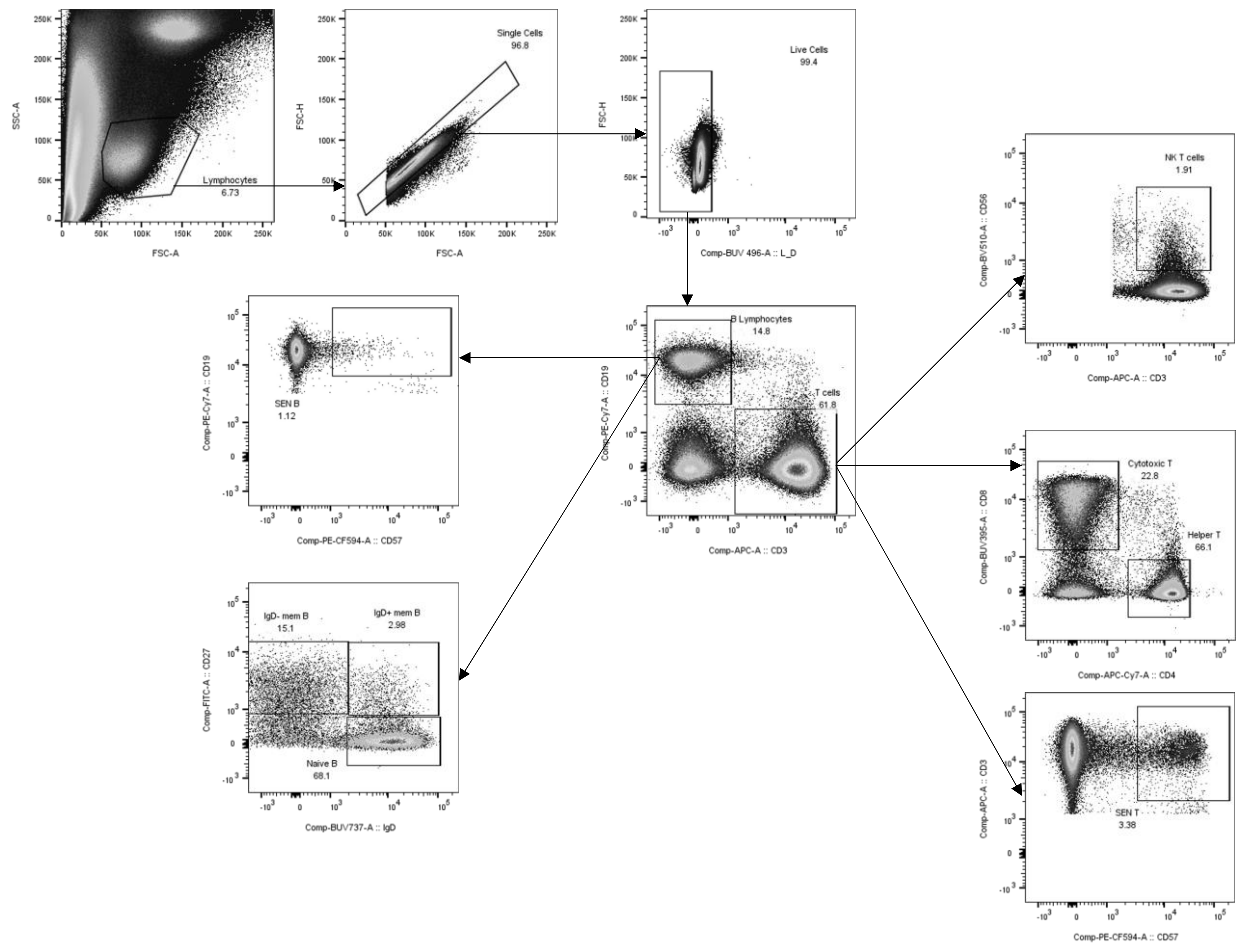

Supplemental Figure S1. Gating strategy for major populations.

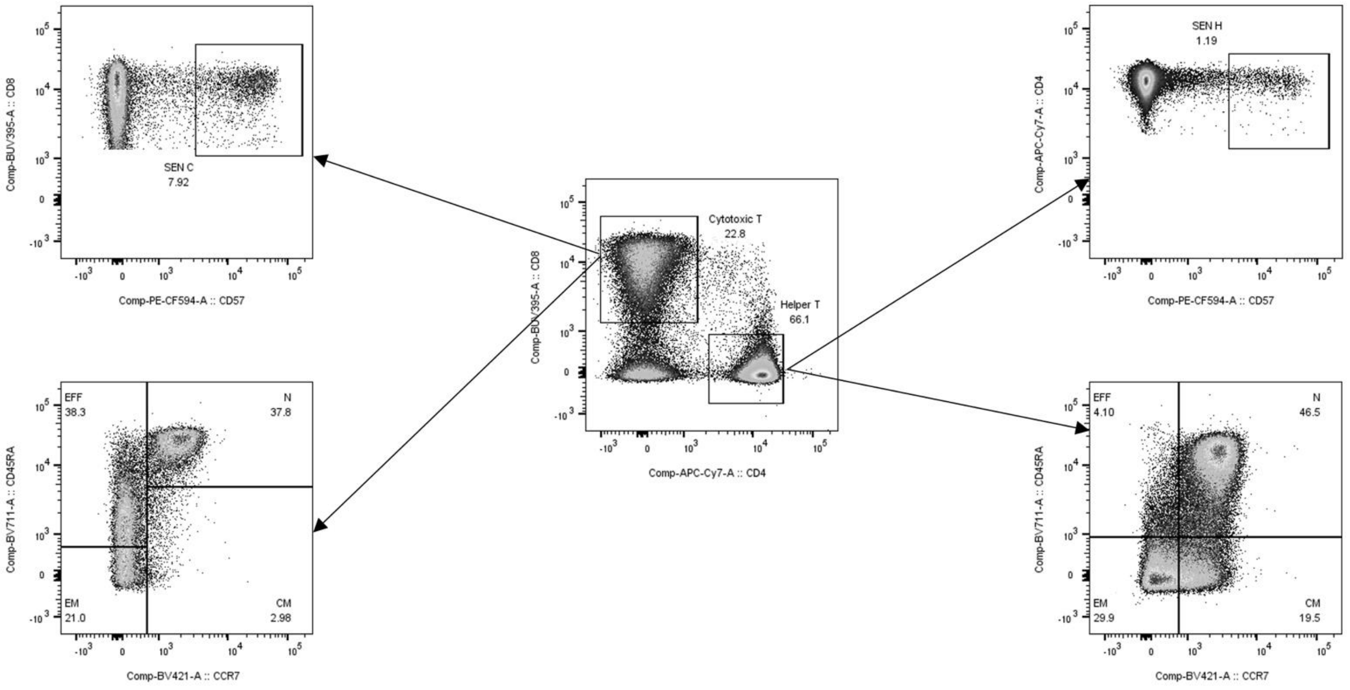

Supplemental Figure S2. Gating strategy for major populations.
